# Family systems care approaches and methodologies for maternal, newborn and child health in low- and middle-income countries: a scoping review protocol

**DOI:** 10.1186/s13643-025-02951-8

**Published:** 2025-10-17

**Authors:** Christina Schuler, Faith Agbozo, George Edward Ntow, Barbara Preusse-Bleuler, Riccardo E. Pfister

**Affiliations:** 1 Institute of Global Health, Faculty of Medicine, University of Geneva, Geneva, Switzerland; 2 Institute of Nursing, School of Health Sciences, Zurich University of Applied Sciences (ZHAW), Winterthur, Switzerland; 3 Department of Family and Community Health, Fred N. Binka School of Public Health, University of Health and Allied Sciences, Ho, Ghana; 4 Dodowa Health Research Centre, Dodowa, Ghana; 5 Neonatal and Paediatric Intensive Care Unit, University Hospitals of Geneva and Geneva University, Geneva, Switzerland

**Keywords:** Continuity of patient care, Family centred care, Maternal, newborn and child health, Health personnel, Hospital to home transition, Low- and middle-income countries

## Abstract

**Introduction:**

Providing family-centred maternal, newborn and child health (MNCH) services along a care continuum could significantly improve outcomes in low- and middle-income countries (LMICs). In LIMCs, family systems care (FSC) is currently not well integrated into the continuum of care from hospital, community, to the home levels.

**Objective:**

This scoping review aims to explore the approaches, methodologies and tools used to translate family systems care programmes/interventions in the continuum of care for MNCH in different care settings in LMICs.

**Eligibility criteria:**

This scoping review will include evidence of approaches, methodologies and tools used to translate FSC into the care continuum for MNCH in LMICs. It will also include evidence reporting on contextual determinants, such as mitigating and limiting factors. Evidence not in English, outside the MNCH scope or using a person- rather than family-centred approach will be excluded.

**Methods:**

Following the recommendations of the Joanna Briggs Institute and the PRISMA Extension for Scoping Review checklist, the following stages will involve two or more researchers: (1) literature search, (2) data extraction, (3) analysis and (4) narrative summary. Medical Literature Analysis and Retrieval System Online (Medline), Cumulative Index to Nursing and Allied Health Literature (CINAHL), Web of Science and grey literature via organisational homepages will be searched. Published English work without limitation on publication year is eligible. Data extraction and analysis will be guided by a template comprising approaches, methodologies and tools to translate FSC into the care continuum. Data will be presented in tabular form with an accompanying narrative summary.

**Systematic review registration:**

The protocol is registered with the Open Science Framework (https://osf.io/zwg9k).

## Introduction

To reduce the risk of maternal, newborn and child mortality, morbidity and disability, the availability, affordability and quality of maternal, newborn and child healthcare (MNCH) services are critical [1]. Providing MNCH in a continuum of care (CoC) could significantly improve MNCH outcomes, particularly in low- and middle-income countries (LMICs) [2]. In addition, the economic burden may even be reduced through high-quality structured and standardised care within a well-coordinated CoC [3, 4]. An effectively organised CoC ideally links home and community (primary) care to hospital care [5]. The CoC concept was first described by Liebowitz and Brody [6] as an integrated service with appropriate health personnel to deliver it. Kerber et al. [5] adapted the CoC for MNCH services in the context of resource-limited countries. They emphasised the importance of interconnectedness among different packages of MNCH. The maternal health package refers to women’s health during pregnancy, childbirth and the postpartum period [7]. Newborn health includes the care for every newborn, including small and sick newborns, from birth up to the first month of life [8]. The child health package contains health services for all children, including children with disabilities and sick or injured children [9]. Maternal, newborn and child health services can be provided in outpatient clinics, facility-based settings or through home care. Emphasis is placed on the connections between components along the continuum of care, encompassing people, places and times [10].

An effectively coordinated care process involves families, empowers them and places them at the centre of the care process [11–13]. Although quality family-centred care has the potential to minimise the risk of mortality, morbidity and disability, families are rarely involved in MNCH services provided at the hospital, community and home levels [14–16]. Often, only mothers and their children are targeted and not the family as a whole [2].

A way to involve families in the caregiving of their newborns and children from pregnancy through birth, postnatal care to childhood, is through family systems care (FSC). FSC facilitates tailored assessments and ongoing support for physical and psychosocial needs by considering the human being as a physical and mental being within their eco-social living environment [17]. FSC extends the concept of family-centred care [18]. Both concepts view parents, families and health professionals as mutually beneficiary partners revolving around principles of dignity and respect, information sharing, shared decision-making and collaboration [11]. In this research, we use the term FSC to refer to all these concepts, including family systems nursing, family-focused care and family engagement.

Recent studies have shown that women and families with low birth weight infants, premature infants or sick children need diverse support, including psychosocial, practical (household chores), material and spiritual support to facilitate effective care of their infants in the hospital and also at home [19–21]. Health professionals, particularly nurses and midwives, are uniquely positioned to provide evidence-based information and psychological support and to empower families to cope with illness and suffering [22–24].

FSC, combined with CoC, aims to provide comprehensive care addressing medical needs and emotional and social support to families [1]. It ensures that families receive consistent assistance and information from pregnancy through childhood [25].

Although this combination has received limited attention thus far, it stresses the importance of providing holistic care to families across the continuum of care. It considers the family’s history, present circumstances and future needs while fostering a trusted relationship and mutual understanding and building family-tailored interventions involving health professionals and family members [26, 27].

Translating FSC into practice is a complex process, and sustaining it is challenging [28, 29]. It necessitates robust research methods and implementation approaches [28, 30]. Knowledge of implementation strategies of FSC programmes in real-world settings, especially in low- and middle-income countries (LMICs), remains limited [31–33].

Little is reported on implementing the combination of FSC and CoC in MNCH, including the lack of summarised data and limited published studies, programmes and interventions. We conducted a preliminary search in PubMed, the Cochrane Database of Systematic Reviews and Joanna Briggs Institute (JBI) Evidence Synthesis and found no published or ongoing systematic or scoping reviews on this topic.

Given the current lack of synthesised evidence on contextual determinants, we consider a scoping review the most appropriate approach to map the existing literature. Scoping reviews are particularly suited to complex and heterogeneous topics where the evidence is diverse and a more focused systematic review would be premature or too narrow [34, 35].

This scoping review aims to comprehensively explore, identify and map the extent of literature on approaches, methodologies and tools used to translate FSC programmes/interventions in the CoC for MNCH into different care settings in LMICs. Findings from this scoping review can inform the future implementation of FSC into the maternal, newborn and child health continuum of care.

### Review question

Specifically, the review will explore:

1. What family systems care (FSC) interventions/programmes have been integrated into the continuum of care (CoC) for maternal, newborn and child health (MNCH) in low- and middle-income countries (LMICs) and what approaches, methodologies and tools have been used?
2. What contextual determinants, including mitigating and limiting factors, affect the uptake of FSC practices and effective translation of interventions/programmes across the CoC for MNCH in LMICs?
3. Which family members and health care professionals are involved in FSC interventions/programmes across the CoC for MNCH in LMICs?

### Eligibility criteria

Following the Joanna Briggs Institute (JBI) guidance [35], the inclusion and exclusion criteria are categorised and defined in terms of participants, concept, context (PCC) and sources (see Table 1) [36]. Articles published in English without a time limit are eligible. Grey literature will be included to increase the comprehensiveness of available evidence in this scoping review [37], as ongoing programmes from organisations that are made publicly available on their web pages are not always converted into publications.

**Table 1:** Eligibility criteria

	Defining characteristics
**Inclusion criteria**	
Participants	*Family members* using MNCH services, families with children up to the age of 12, women using maternal health services, parents, siblings, grandmothers, grandfathers, uncles, aunts and other significant caregivers *Health professionals*: nurses, midwives, advanced practice nurses and midwives, physician assistants, doctors *Lay workers:* e.g. community health volunteers, traditional birth attendants delivering MNCH services
Concept	Family systems/care/nursing/centred care, family engagement intervention and programmes, approaches, methodologies, tools, contextual determinants including enabling and limiting factors for successful translation
Context	Studies should encompass the care continuum, including settings such as primary care/community, home and hospital/health facilities and outpatient clinics Low- and middle-income countries
Type of sources	Any existing literature, including journal articles, and grey literature, including information on webpages and evaluation reports
Time period	No time restriction
Language	English
**Exclusion criteria**^a^	
Reason for exclusion	Articles written in languages other than English Studies not within the scope of MNCH Studies with a person-centred approach (not family-centred) aiming at one person alone Not meeting the inclusion criteria listed above

^a^*MNCH *Maternal, newborn, and child health

### Participants

This scoping review will include two groups of participants. The first group will be families of patients using MNCH services. We will apply the term family as it has been defined by the Vanier Institute of the Family [38]: ‘Any combination of two or more persons who are bound together over time by ties of mutual consent, birth and/or adoption or placement, and who together assume responsibilities for variant combinations of some of the following: physical maintenance and care of group members; addition of new members through procreation, adoption or placement; socialisation of children; social control of members; production, consumption, distribution of goods and services; and affective nurturance (i.e. love).’ Therefore, we will include women who use maternal health services (e.g. pregnant women), mothers, fathers, siblings, grandparents and other relatives or any significant caregivers or support persons such as family, friends and neighbours WHO are involved in the care process. Families with children up to 12 years old will be considered. The second target group includes health professionals and lay workers delivering MNCH services. Health professionals may include nurses, midwives, advanced practice nurses and midwives, physician assistants and doctors. Lay workers can include community volunteers and traditional birth attendants delivering some services to families in the field of MNCH.

### Concept

This review explores the concept of family systems care (FSC). This will include studies using the terms family systems care, family-centred care, family nursing care, family engagement and/or family structured care. Publications that report on methodologies, approaches and tools used to translate an FSC programme/intervention into MNCH and contextual determinants that influence this translation will be included. Studies on FSC that focus on other areas outside the field of MNCH, such as family systems care for adults or elderly people, will be excluded.

### Context

Programmes and interventions must occur in the care pathways between hospital, community and home settings, including the discharge, referral or transfer procedures. Ideally, articles should have reported on the transitions between the different levels of care. Studies conducted in low- and middle-income countries (LMICs) will be considered. The categorisation for LMICs published by the World Bank [39] will be used.

### Type of sources

This scoping review will consider both quantitative and qualitative studies. Study designs, such as experimental and quasi-experimental study designs, including randomised controlled trials, non-randomised controlled trials, before and after studies and interrupted time-series studies, will be included. Analytical observational studies, including prospective and retrospective cohort studies, case-control studies and analytical cross-sectional studies, will be considered for inclusion. This review will also include descriptive observational study designs, case series, individual case reports and descriptive cross-sectional studies. Qualitative studies will consider designs such as phenomenology, grounded theory, ethnography, qualitative description, action research, case study research and feminist research. Grey literature, such as reports from organisations, policy documents and unpublished studies that carry out programmes with an FSC approach but have not been published in scientific journals, will also be deemed eligible if the description of the methods used is detailed and helpful to answer the research questions.

## Methods

A scoping review approach was chosen due to the broad nature of our research question. ‘Scoping reviews are helpful when the literature in an area is complex and heterogeneous and is not amenable to a more precise systematic review of the evidence’ [34, 35]. It is an ideal tool to determine the scope or coverage of a body of literature on a given topic and clearly indicate the volume of literature and studies available as well as an overview (broad or detailed) of its focus [40]. The proposed scoping review will be conducted following the recommendations of the Joanna Briggs Institute (JBI) methodology for scoping reviews [35]. The reporting of this scoping review will follow the most current version of the Preferred Reporting Items for Systematic Reviews and Meta-Analyses extension for Scoping Reviews (PRISMA-ScR) available at the time of submission [41, 42]. Any amendments to the scoping review that differ from the methods outlined in this protocol will be clearly documented and reported in the final review.

### Search strategy

The search strategy will aim to locate both published and unpublished primary studies online, such as journal articles and grey literature, including information on webpages and evaluation reports. The search strategy has been developed with the support of a research librarian who constructed expert searches tailored to each database. An initial limited search of Medical Literature Analysis and Retrieval System Online (Medline) and Cumulative Index to Nursing and Allied Health Literature (CINAHL) was undertaken to identify articles on the topic. The initial search strategy for Medline via Ovid is reported in Appendix 1. The text words contained in the titles and abstracts of relevant articles, as well as the index terms used to describe the articles, will be used to develop a complete search strategy. The final search will be done in Medline (Ovid), CINAHL (Ebsco), Web of Science Core collection and grey literature from specific organisational websites. Besides reviewing the reference lists of included articles, the search strategy, including all identified keywords and indexed terms, will be adapted for each included database and information source. Sources of unpublished studies/grey literature will be searched via the organisation homepages from African Medical and Research Foundation (AMREF), Care International, Enfants du Monde, Family Health International 360 (FHI 360), Healthy Newborn Network (HNN), Inter-Agency Working Group on Reproductive Health in Crises (IAWG), IBP Network, International Committee of the Red Cross (ICRC), International Rescue Committee (IRC), Johns Hopkins Program for International Education in Gynaecology and Obstetrics (Jhpiego), John Snow, Inc. (JSI), Management of At-risk Mothers and Infants under six months (MAMI) Global Network, Marie Stopes International (MSI), Médecins Sans Frontières (MSF), Newborn Essential Solutions and Technologies 360 (NEST 360), newborntoolkit.org, PATH, Population Council, RAISE Initiative, Results for Development (R4D), Resilient, Inclusive, Sustainable Education (RISE 360), Save the Children, Solidarmed, SickKids for Global Health, United Nations International Children’s Emergency Fund (UNICEF), United Nations Population Fund (UNFPA), USAID Momentum and Women’s Refugee Commission (WRC).

A secondary search to identify recently published articles will be performed before completing this scoping review.

### Source of evidence selection

Following the search, all identified citations will be collated and uploaded into the newest EndNote version (Clarivate Analytics, PA, USA) and duplicates will be removed using its automatic function. References will be transferred to Rayyan software (Qatar Computing Research Institute, Doha, Qatar) [43] to screen titles and abstracts. Two independent reviewers will conduct a pilot test of 10 to 15 articles to assess the reviewer agreement regarding the relevance of titles and abstracts. Following the pilot test, titles and abstracts will be screened and assessed by at least two independent reviewers against the review’s eligibility criteria. Potentially relevant sources will be retrieved in full, and their citation details will be imported into the Rayyan software. The full text of selected citations will be assessed in detail against the inclusion criteria by two or more independent reviewers. The scoping review will record and report reasons for excluding articles at the full-text stage. Any disagreements that arise between the reviewers at each stage of the selection process will be resolved through discussion or with an additional reviewer to find consensus.

The targeted organisational homepages will be searched for potentially relevant information considering our eligibility criteria. The same keywords and search terms will be used. If titles or subtitles seem relevant, they will also be uploaded into the Rayyan software and screened by two independent reviewers.

Within each of these search methods, ‘snowball’ searching will also be applied if any records appear to be relevant (i.e. using reference lists from included articles). Snowballing is an effective way to increase the thoroughness of search results and the number of included papers in reviews [44]. Reviewers will have regular meetings during the screening process of titles and abstracts, full texts and data extraction to discuss and resolve emerging issues. The unit of analysis for this scoping review will be any individual primary study or report. We will adopt the definitions of ‘study’ and ‘report’ as outlined in the PRISMA 2020 guidelines for systematic reviews to ensure consistency and clarity [45]. A *study* refers to an investigation (e.g. a clinical trial or observational study) involving a defined group of participants and one or more interventions and outcomes. A single study may give rise to multiple *reports*, such as a protocol, statistical analysis plan or separate publications for different outcomes. A *report* is any document (published or unpublished) that provides information about a particular study, including journal articles, preprints, conference abstracts, registry entries, dissertations, clinical study reports or government documents [45].

The results of the search strategy and selection process will be reported in full in the final scoping review and presented in the PRISMA-ScR flow diagram [41].

### Data extraction

Data will be extracted from papers included in the scoping review by two or more independent reviewers using a data extraction tool developed by the reviewers (see Appendix 2). The data extracted will include specific details about the participants, concept, context, study designs, study methods, tools used and key findings relevant to the review questions. The draft data extraction tool has undergone pretesting and is ready for use in its current form. Any disagreements that arise between the reviewers will be resolved through discussion or with an external reviewer. If appropriate or required, authors of papers will be contacted to request missing or additional data.

### Data analysis and presentation

The evidence will be analysed and presented through graphs and summary tables (see Appendix 2). First, a frequency count of identified articles or reports published on family systems care approaches along the care continuum for MNCH in low- and middle-income countries (LMICs) will be performed.

We will then report on publication details like authors and year of publication, title, country of study/programme and year of data collection. Details of involved family members, health professionals and lay workers will follow. Information about the concept and context will be reported on. Details of the evidence source, such as study design, methods, objectives and implementation details, as well as enabling and limiting factors, will be described. The content of the FSC programme used will be reported as well.

The tables and figures will include text that provides details and rich descriptions of its content. A narrative summary describing how results relate to the review’s objectives will then be generated.

According to the Joanna Briggs Institute (JBI) methodology, a systematic appraisal of the methodological quality of studies is not required in a scoping review [35, 36] and will, therefore, not be done.

## Data Availability

Not applicable.

## Appendix 1

### Search strategy

**Table 2:** Search strategy for Medline via Ovid and initial hints

**#**	**Query**	**Results from 28 May 2023**
1	FAMILY NURSING/	1,568
2	FAMILY SUPPORT/	50
3	FAMILY/	84,481
4	1 or 2 or 3	85,861
5	(“FAMILY CENTRED CARE” or “FAMILY CENTERED CARE” or FAMILY or “FAMILY NURSING” or “FAMILY FOCUSED CARE” or “FAMILY SYSTEMS NURSING” or “FAMILY SYSTEMS APPROACH” or “FAMILY PARTICIPATORY CARE” or “family adj2 CARE”).ab,ti,tw.	930,552
6	4 or 5	969,864
7	CONTINUITY OF PATIENT CARE/	20,629
8	HOSPITAL TO HOME TRANSITION/	45
9	PATIENT DISCHARGE/	39,269
10	PATIENT HANDOFF/	1,577
11	TRANSITIONAL CARE/	1,231
12	7 or 8 or 9 or 10 or 11	59,687
13	(“CARE CONTINUUM” or “CONTINUITY OF CARE” or “CARE CONTINUITY” or “PATIENT CARE CONTINUITY” or “CARE PATHWAY” or “REFERRAL PATHWAY” or “REFERRAL COMPLETION” or DISCHARGE).ab,ti,tw.	251,444
14	12 or 13	278,069
15	MATERNAL HEALTH/	2,280
16	MATERNAL HEALTH SERVICES/	16,292
17	PREGNANCY/	983,608
18	15 or 16 or 17	988,196
19	(“PRENATAL CARE” or “ANTENATAL CARE” or “INTRAPARTUM CARE” or PREGNANCY or “MATERNAL CARE” or “POSTPARTUM PERIOD” or “CHILD BIRTH”).ab,ti,tw.	464,029
20	18 or 19	1,086,261
21	INFANT/	864,234
22	INFANT HEALTH/	1,260
23	21 or 22	864,898
24	(“HEALTH OF NEWBORN INFANTS” or “HEALTH OF THE NEWBORN INFANT” or “NEWBORN INFANT HEALTH” or “BABY HEALTH” or “NEONATAL HEALTH” or “NEWBORN HEALTH” or “POSTNATAL CARE” or “NEWBORN CARE” or NEWBORN).ab,ti,tw.	140,181
25	23 or 24	983,903
26	CHILD/	1,906,398
27	CHILD HEALTH/	4,969
28	CHILD-HEALTH SERVICES/	21,339
29	MATERNAL-CHILD HEALTH SERVICES/	959
30	26 or 27 or 28 or 29	1,914,095
31	(“CHILDREN’S HEALTH” or “CHILD HEALTH” or “CHILD CARE” or CHILDREN or “CHILD WELL BEING” or “CHILD WELLBEING” or “CHILD HEALTH SERVICES” or “YOUNG CHILDREN”).ab,ti,tw.	1,221,485
32	30 or 31	2,347,575
33	DEVELOPING COUNTRIES/	80,799
34	(“DEVELOPING COUNTRIES” or “DEVELOPING NATIONS” or “DEVELOPING NATION” or “LEAST DEVELOPED COUNTRY” or “LESS DEVELOPED COUNTRY” or “LESS DEVELOPED COUNTRIES” or “THIRD WORLD COUNTRIES” or “THIRD WORLD COUNTRY” or “THIRD WORLD NATION” or “UNDER-DEVELOPED COUNTRY” or “LOWER MIDDLE INCOME COUNTRY” or “LOWER-MIDDLE-INCOME COUNTRIES”).ab,ti,tw.	71,621
35	33 or 34	133,514
36	6 and 14 and 20 and 35	34
37	6 and 14 and 25 and 35	15
38	6 and 14 and 32 and 35	28

## Appendix 2

### Data extraction instrument

**Table:**

Publication details				Participants		Concept	Context
Authors and year of publication	Title	Journal and DOI	Country of study/program Year of data collection	Type of family members	Type of health professionals

**Table:**

Publication details	Evidence source details and characteristics						
Authors and year of publication	Research design	Methods details	Objectives	Implementation methodologies/approaches/tools	Enabling factors	Limiting factors	Content of FSC

## Abbreviations

CoC: Continuum of care
FSC: Family systems care
JBI: Joanna Briggs Institute
LMICs: Low- and middle-income countries
MNCH: Maternal, newborn and child health
PCC: Participant, concept, context
PRISMA-ScR: Preferred Reporting Items for Systematic Reviews and Meta-Analyses Extension for Scoping Reviews
